# Comparison of intramuscular versus subcutaneous aqueous progesterone for luteal phase support in artificially prepared frozen embryo transfer cycles

**DOI:** 10.4274/tjod.galenos.2020.01460

**Published:** 2020-12-10

**Authors:** Emre Niyazi Turgut, Fazilet Kübra Boynukalın, Meral Gültomruk, Zalihe Yarkıner, Mustafa Bahçeci

**Affiliations:** 1 Bahçeci Health Group, Fulya IVF Center, İstanbul, Turkey; 2 Cyprus Science University, Fakulty of Medrome, Department of Statistics, Kyrenia, Cyprus

**Keywords:** Subcutaneous aqueous progesterone, intramuscular progesterone, artificially prepared frozen embryo transfer

## Abstract

**Objective:**

Cryopreservation of embryos for future transfer attempts has noticeably increased in the last decade, especially due to the technological developments in in vitro fertlization (IVF) laboratories. In parallel, different progesterone (P) replacement regimens preceding artificially prepared frozen embryo transfer (AC-FET) attempts, especially with respect to the route of application and dosing scheme, have been widely argued so far. We aimed to provide more information about the efficacy profile of novel subcutaneous aqueous progesterone (SP) in AC-FET cycles.

**Materials and Methods:**

This retrospective, single-centre cohort study included a total of 507 AC-FET cycles performed between June 2018 and April 2020. Three hundred forty-nine (68.8%) patients received 50 mg of intramuscular progesterone as once daily, 158 (31.2%) patients received 25 mg of SP as twice daily. Only, the first and single blastocyst transfers from the same cohort were accepted. The inclusion criteria were as follows: females aged <37 years, body mass index ≥18 kg/m^2^ and ≤35 kg/m^2^, sperm concentration ≥5x10^6^/mL. Pre-implantation genetic testing cycles were not included. The primary outcome was the live birth rate (LBR).

**Results:**

The number of previous IVF attempts, type of infertility, peak estradiol (E2) levels, the total number of retrieved oocytes, mature oocytes, and the number of 2PN was significantly different between the groups. Positive pregnancy (p=0.474) and clinical pregnancy rates (p=0.979), LBR (p=0.404), and missed abortion rates (p=0.144) were comparable between the groups. The total number of oocytes [adjusted odds ratios (AOR)=1.024, 95% confidence interval (CI): 1.002-1.047; p=0.03)], endometrial thickness (AOR=1.121, 95% CI: 1.003-1.253; p=0.044), and cryopreservation day 5/6 (AOR=0.421, 95% CI: 0.226-0.788; p=0.007) achieved statistical significance following binary logistic regression analysis. However, P administration type did not achieve statistical significance (p=0.731).

**Conclusion:**

As a novel option, SP has comparable efficacy in pregnancy outcomes and may be accepted as an alternative for luteal phase support in AC-FET cycles.

**PRECIS:** Subcutaneous aqueous progesterone is an effective alternative to intramuscular progesterone in artificially prepared frozen embryo transfer cycles.

## Introduction

Almost 37 years ago, the first human pregnancy was reported following frozen embryo transfer (FET)^(1)^. Following the developments in the *in vitro* fertilization (IVF) field, the cryopreservation of embryos and subsequent FET strategy has doubled in the last decade^(2)^. Artificial endometrial preparation is one of the methods used for FET cycles and has been found as successful as the other approaches^(3)^. In these cycles, minimal monitoring is required, and the timing of embryo transfer and initiation of progesterone (P) is more flexible^(4)^. Therefore, it allows both the physicians and embryology staff to easily organize daily business planning.

Exogeneous P replacement is preceded by estrogen supplementation and its use is mandatory to prepare the endometrium for successful implantation and the survival of the pregnancy^(5)^. Exogeneous P can be administered by different routes: intramuscular, vaginal, oral, rectal, and recently, subcutaneous. Oral micronised P formulations are exposed to the first-pass effect within the liver, hence they have a low effect profile^(6)^. Vaginal formulations such as capsules, gels or suppositories showed a similar efficacy profile when compared with each other or by the intramuscular route^(7,8,9)^. However, debates regarding the method of application, the timing for luteal phase support (LPS), and doses are ongoing^(10)^. Oil-based intramuscular progesterone (IMP) preparations are painful and may cause serious adverse effects such as skin inflammation and sterile abscesses, but they have been found to decrease subendometrial uterine contractility better than vaginal progesterone (VP), and this positive effect has been related to increased pregnancy outcomes and decreased rates of embryo displacement following the attachment process^(11)^.

In the light of new technological developments, subcutaneous aqueous progesterone (SP) has gained a more hydro-soluble and absorbable state by the addition of β-cyclodextrin^(12)^. Two randomized controlled trials (RCT) conducted on fresh transfer cycles compared the efficacy of SP and VP and reported similar ongoing pregnancy rates (OPRs) and live birth rates (LBRs)^(13,14)^. Regarding the degree of acceptance and satisfaction, the authors found significantly increased acceptance rates for the SP route compared with VP^(15)^.

The use of SP continues to gain popularity in our daily practice. Today, most physicians use VP or IMP regimens, alone or in combination, for LPS. Currently, there are insufficient data on the effectiveness of the new formulation in artificially prepared FET (AC-FET) cycles. Therefore, in our study, we aimed to contribute to the literature by comparing two different P replacement regimens, SP vs. IMP, in elective, single blastocyst AC-FET cycles.

## Materials and Methods

### Design

In this retrospective, single-center cohort study, we reviewed the pregnancy outcomes of 507 AC-FET cycles, performed between June 2018 and April 2020 in Bahceci Fulya IVF centre. The reason for choosing the time interval in this way was the introduction of the SP formulation in Turkey in June 2018. Ethics approval was obtained from the institutional review board (approval number: 59, date: 21/03/2020). Based on our experience and reports in the literature, we switched to the freeze-all and subsequent FET strategy in all IVF cycles in 2013 due to its superior reproductive outcomes compared with fresh transfer^(16,17)^. Only the first single blastocyst transfers from the same cohort were included in the study. The eligibility criteria for the couples were as follows: female age <37 years, body mass index (BMI) ≥18 kg/m^2^ and ≤35 kg/m^2^, sperm concentration ≥5x10^6^. Couples with a history of repeated implantation failure (>2), recurrent miscarriages (≥2), past surgery(-ies) for intrauterine adhesions, submucosal fibroids and mullerian anomalies (unicornuate, bicornuate, septate uterus) were excluded from the study. Also, couples carrying chromosomal abnormalities and preimplantation genetic testing cycles were not included.

### Ovarian Stimulation

The gonadotropin-releasing hormone (GnRH) antagonist protocol was the preferred method for ovarian stimulation. On the 2^nd^ or 3^rd^ day of the menstrual cycle, gonadotrophin injections were started by using recombinant follicle-stimulating hormone (Gonal-F; Merck Serono, Geneva, Switzerland) and/or highly purified human menopause gonadotrophins (hp-hMG) (75-150 IU, Merional; IBSA) preparations. The dose regimens were designated at the physician’s preference. When the leading follicle exceeded 13 mm in diameter, 0.25 mg of GnRH antagonist (Cetrotide; Serono) was started daily until the day of maturation trigger. Maturation of the oocytes was induced either with the use of 250 µg of human chorionic gonadotropin (hCG; Ovitrelle, Serono) or 0.2 mg triptorelin (Gonapeptyl, Ferring). Transvaginal sonography (TV-USG)-guided oocyte retrieval was performed 35-36 hours later.

### Laboratory Process

After the denudation process, each metaphase II oocyte was injected with sperm using the intracytoplasmic sperm injection technique and cultured individually in a special pre-equilibrated culture dish. A fertilization check was performed 16-18 hours after insemination. A single-step media (Irvine Scientific, CA, USA) was used throughout the blastocyst culture period. Blastocyst quality assessment was performed on day 5 or 6 by two senior embryologists, with the aid of a morphology-based three-part scoring system as described previously^(18,19)^. Once the embryo reached the expansion degree of at least 3, vitrification was performed for cryopreservation. Categorization of blastocysts was as follows: excellent (≥3 AA), good (3, 4, 5, or 6 and AB, AC, BA, BB), poor (3, 4, 5, or 6 and BC, CB, CC, or CA).

### Artificial Preparation of FET Cycle

Endometrial preparation was started on day 2 or 3 of menstrual bleeding with estradiol valerate pills (Estrofem, Novo Nordisk, Denmark) at a dosage of 6 mg/day. A stable dosing scheme was implemented. Follow-up visits were performed between day 10 and 14 of treatment. Endometrial thickness was measured using TV-USG and blood was drawn to detect serum estradiol (E2) and P levels. The dosage of E2 pills was increased to 8 mg/day if the thickness was <7 mm and an additional follow-up visit was planned within the next seven days for confirmation. According to the patient’s and physician’s preference, LPS was initiated either with 50 mg IMP injection (Progestan, Kocak Farma, Turkey) once per day, or with 25 mg of SP (Prolutex, IBSA, Switzerland) injections, twice daily. The first dose of IMP was injected between 4 and 7 pm, and subsequent doses were repeated every 24 hours at the same time interval. For the SP injection, the first dose was injected between 8 am and 10 am, and the second dose was injected 12 hours later. The same scheme was followed every day. In our daily routine, all transfers are performed between 4 pm and 7 pm. Accordingly, FET was performed following the 5^th^ dose of IMP and the 11^th^ dose of SP administration. Serum β-hCG levels were measured 12 days after FET and levels ≥5 IU were accepted as positive. Afterwards, E2 replacement was stopped at the 6^th^ week of pregnancy, whereas P was continued until 10 weeks in both arms.

### Outcomes

Primary outcome was the LBR per embryo transfer. Clinical pregnancy (CP) was defined as the confirmation of an intrauterine gestational sac at 6-7 weeks of pregnancy. Missed abortion (MA) was defined as a CP loss before 20 weeks’ gestation.

### Statistical Analysis

For the first step, the Kolmogorov-Smirnov and Shapiro-Wilk tests were performed to understand whether the continuous variables followed a normal distribution. Accordingly, the median (quartile 1- quartile 3) values of these variables were reported in the tables. Afterwards, the independent samples median test was run to determine if there were differences in continuous parameters between patients in the two treatment groups. The chi-square test was performed to test the significance of each categorical parameter and the results were reported as percentages.

A binary logistic regression model was performed regarding outcomes to determine whether a patient was having a live birth. In this model, female age, duration of infertility, sperm concentration, type of infertility, total number of retrieved oocytes, endometrial thickness, cryopreservation day (D5 or D6), blastocyst quality (excellent, good, poor), peak E2 levels in FET and type of P administration (IMP or SP) were allocated as independent variables. The backward conditional procedure was used and variables that were not statistically significant were removed from the model. The final binary logistic model reported only the statistically significant parameters. To measure the effect of each significant variable, both unadjusted and adjusted odds ratios were reported. Unadjusted odds ratios (UAOR) indicated the effect of each variable when all of the other factors were eliminated and only the specific variable was taken into consideration. Adjusted odds ratios (AOR) were calculated when all the significant independent variables were taken into account, simultaneously.

## Results

All 507 patients in our study were assigned to one of the two LPS alternatives. IMP was used in 349 (68.8%) AC-FET cycles and SP was used in 158 (31.2%) AC-FET cycles. Two groups were matched concerning demographics and embryologic parameters as shown in Table 1. Accordingly, the median values of the number of previous IVF attempts, peak E2 levels, the total number of oocytes, mature oocytes, and the number of 2PN zygotes were significantly different between groups.

Table 2 displays the characteristics of AC-FET cycles and pregnancy outcomes. As shown, the only parameter to reach statistical significance was the peak E2 levels, which were measured on the day of or one day before the initiation of P replacement (p=0.025). There were no significant differences between groups, regarding positive pregnancy rates (p=0.474), CP rates (p=0.979), LBRs (p=0.404), and MA rates (p=0.144).

Binary logistic regression analysis was performed to determine the independent variables, those which had a significant effect on live birth outcome (Table 3). The final model was statistically significant, χ^2^ (2)=18.373, p<0.001. The model explained 4.8% (Nagelkerke R^2^) of the variance in live births and correctly classified 62.1% of cases. As shown in Table 3, UAOR and AOR concluded that variables such as the total number of retrieved oocytes, endometrial thickness, and cryopreservation day were statistically significant both when considered separately and when taken into analysis at the same time. AC-FET cycles using day 6 cryopreserved blastocysts resulted in a 57.9% less likely live births compared with day 5 blastocyst transfers [(AOR=0.421, 95% confidence interval (CI): 0.226-0.788; p=0.007)]. It is also shown that when the total number of retrieved oocytes increased by one unit, patients were 1.024 times more likely to have a live birth, and similarly, when the endometrial thickness increased by one unit, patients were 1.121 times more likely to have a live birth (AOR=1.024, 95% CI: 1.002-1.047; p=0.03 and AOR=1.121, 95% CI: 1.003-1.253; p=0.044, respectively). P administration type did not achieve statistical significance (p=0.731).

## Discussion

As far as we know, this is the first study to compare the clinical efficiency profiles of the novel aqueous SP formulation and IMP in AC-FET cycles. The results of our study showed non-inferior pregnancy outcomes of 50 mg daily SP administration in women undergoing AC-FET compared with IMP.

For many years, owing to its insoluble properties, the only way to administer the synthetic progesterone hormone was through intramuscular injections. Although it has many adverse effects and causes discomfort, most studies used IMP as a reference when comparing other formulations due to its reliable contributions to pregnancy outcomes^(20,21,22,23)^. The aim of producing a new injectable P formulation was to provide the advantage of existing parenteral injection on pregnancy results, and to eliminate its adverse effects, complications, and negative effects on patient comfort^(24,25)^. For this purpose, Sator et al.^(26) ^assessed the bioavailability of the novel SP formulation in comparison with oil-based IMP among postmenopausal and reproductive-aged women. Irrespective of the route of administration (i.m. and s.c.), serum maximum concentrations (C*max*) of SP product were 3-4 times higher than the C*max *of the oily IMP (p<0.001). Moreover, T*max* (time to achieve C*max*) was 7 times shorter in the SP group. Regarding the safety profiles, the authors reported lower frequency and shorter duration of adverse effects, those related to hormonal changes and injection site reactions. In another valuable study, histologic changes caused by two different dosing regimens, 25 mg/daily and 50 mg/daily, of SP were investigated via endometrial sampling^(27)^. The authors reported adequate pre-decidual transformation within the endometrial specimens of the entire cohort and concluded that the new formulation was a valid option for LPS. From the clinical point of view, the narrow BMI range (>19 and <25 kg/m^2^) in the study should be interpreted with caution and further well-designed studies could give more accurate information, especially in overweight and obese women.

There is still no consensus on the best route of P administration for replacement in AC-FET cycles. According to a Cochrane review, there was no significant difference between VP and IMP in terms of CP, MA rates, and LBRs^(28)^. However, the authors declared that the results were insufficient to draw a definite conclusion due to the heterogeneity between the included studies. In a more recent analysis in which VP and IMP were compared in FET cycles, similar pregnancy outcomes were reported^(8,29)^. By contrast, Devine et al.^(30)^ reported decreased OPRs only in the VP group when compared with VP plus IMP and IMP only, and they terminated the randomization arm due to increased SA rates (47%, 30%, and 23%, respectively, p<0.001). The broad range of age selection criteria (18-48 years) and the nine-day use of VP before FET should be taken into account. In another study, significantly lower rates of CP and live births were reported in the VP group following day 3 FET.

The main limitations of the study were the use of the slow freezing technique for cryopreservation and the 3^rd^ day embryo transfers instead of blastocyst-stage transfers. Similar to the inconsistent results mentioned in the above studies, using oral dydrogesterone for FET cycles also needs further investigation^(31,32)^.

As all IVF practitioners know, daily gonadotropin injections are made throughout the stimulation phase of IVF treatments. Therefore, patients are familiar with subcutaneous injection attempts and feel safe while self-administering SP^(12)^. Moreover, the lesser injection site pain is an advantage of SP, probably related to its water-soluble content^(15)^. Another advantage of SP use is preventing the messy discharge reported with VP application.

### Study Limitations

The major weaknesses of our study are its retrospective design and lack of randomization for the type of P formulations. Its retrospective nature is also the greatest obstacle to reaching information about patient comfort. The main reason for the small sample size is that the SP form started to be used in our country approximately two years ago. We designed this study in patients who were aged younger than 37 years to alleviate the risk of aneuploidy, which might give rise to increased rates of abortions. Four hundred eighty out of 507 (94.6%) patients in the study were aged younger than 37 years. Due to the legal restrictions in our country, we included only single blastocyst transfers. We believe that the strict inclusion and exclusion criteria helped us to generate homogenous groups and detailed analysis of the variables added strength to our work.

## Conclusion

This study provides clinical evidence that the newly developed SP formulation has a comparable efficiency profile on pregnancy outcomes and is a strong candidate for LPS in AC-FET cycles. Future prospective studies and RCT are needed to clarify the best way regarding various P replacement regimens.

## Figures and Tables

**Table 1:**
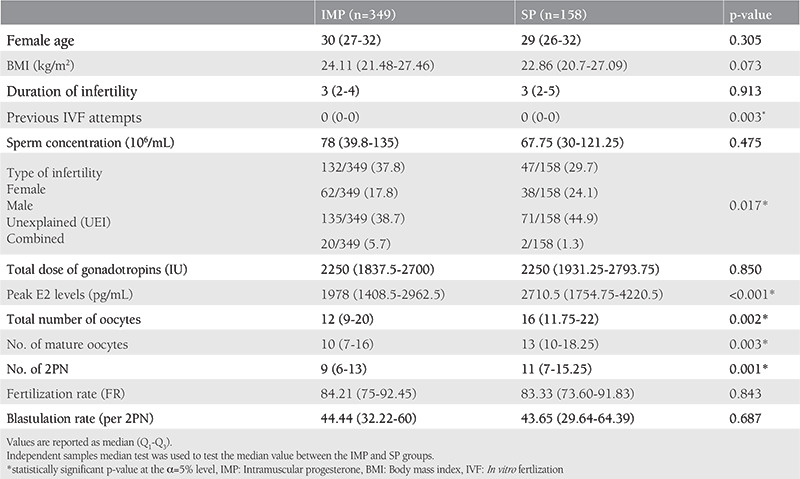
Demographics, clinical and embryologic parameters

**Table 2:**
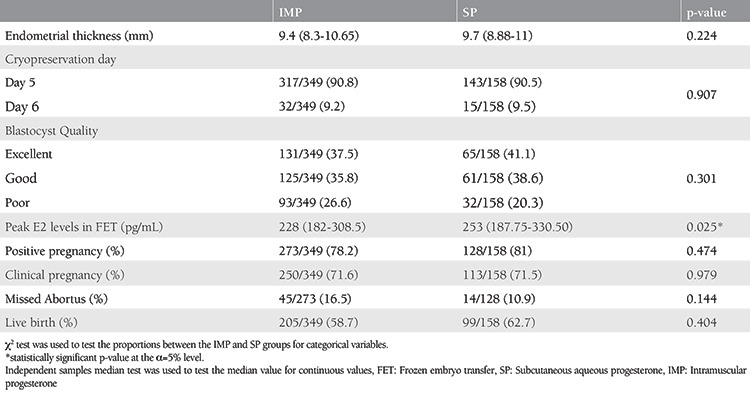
Properties of FET cycles and pregnancy outcomes

**Table 3:**
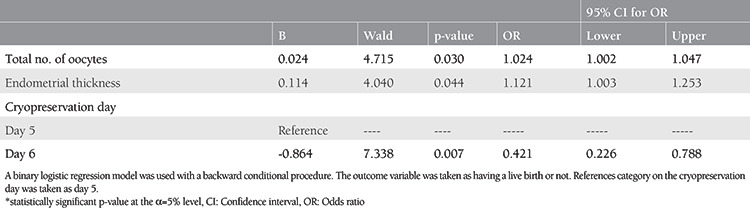
Logistic regression model on live birth outcome
